# Corrigendum: Patients with schizophrenia fail to up-regulate task-positive and down-regulate task-negative brain networks: an fMRI study using an ICA analysis approach

**DOI:** 10.3389/fnhum.2013.00231

**Published:** 2013-05-30

**Authors:** Merethe Nygård, Tom Eichele, Else-Marie Løberg, Hugo A. Jørgensen, Erik Johnsen, Rune A. Kroken, Jan Ø. Berle, Kenneth Hugdahl

**Affiliations:** ^1^Department of Biological and Medical Psychology, University of BergenBergen, Norway; ^2^Section for Clinical Neurophysiology, Haukeland University HospitalBergen, Norway; ^3^Department of Biomedicine, K.G. Jebsen Centre for Research on Neuropsychiatric Disorders, University of BergenBergen, Norway; ^4^Mind Research NetworkAlbuquerque, NM, USA; ^5^Division of Psychiatry, Haukeland University HospitalBergen, Norway; ^6^Section of Psychiatry, Department of Clinical Medicine, University of BergenBergen, Norway; ^7^Department of Radiology, Haukeland University HospitalBergen, Norway

p. 4, “MR IMAGING” section, line 20 from bottom:

The following sentences

“[…] The T1-images were acquired with an EPI sequence. The T1-images were acquired with a FSPGR pulse sequence with 122 sagittal slices [64 × 64 matrix size, slice thickness = 1.0 mm, echo time (TE) = 30 ms, repetition time (TR) = 1.5 s, flip angle (FA) = 90]. […]”

should be replaced by

“[…] The T1-images were acquired with a FSPGR pulse sequence with 180 sagittal slices [256 × 256 matrix size, field of view (FoV) = 256 × 256 mm^2^, slice thickness = 1.0 mm, inversion time (TI) = 500 ms, flip angle (FA) = 11]. […]”

